# The Contribution of High-Order Metabolic Interactions to the Global Activity of a Four-Species Microbial Community

**DOI:** 10.1371/journal.pcbi.1005079

**Published:** 2016-09-13

**Authors:** Xiaokan Guo, James Q. Boedicker

**Affiliations:** 1 Department of Physics, University of Southern California, Los Angeles, California, United States of America; 2 Department of Biological Sciences, University of Southern California, Los Angeles, California, United States of America; University of Wisconsin-Madison, UNITED STATES

## Abstract

The activity of a biological community is the outcome of complex processes involving interactions between community members. It is often unclear how to accurately incorporate these interactions into predictive models. Previous work has shown a range of positive and negative metabolic pairwise interactions between species. Here we examine the ability of a modified general Lotka-Volterra model with cell-cell interaction coefficients to predict the overall metabolic rate of a well-mixed microbial community comprised of four heterotrophic natural isolates, experimentally quantifying the strengths of two, three, and four-species interactions. Within this community, interactions between any pair of microbial species were positive, while higher-order interactions, between 3 or more microbial species, slightly modulated community metabolism. For this simple community, the metabolic rate of can be well predicted only with taking into account pairwise interactions. Simulations using the experimentally determined interaction parameters revealed that spatial heterogeneity in the distribution of cells increased the importance of multispecies interactions in dictating function at both the local and global scales.

## Author Summary

Many wild microbial ecosystems contain hundreds to thousands of species, suggesting that interactions between species likely play an important role in regulating the behavior of such complex cellular networks. Predicting how these interactions impact the overall activity of microbial communities remains a challenge. Here we quantify the contribution of interactions between more than two species to the overall metabolic rate of a mixture of four freshwater bacteria. We systematically measure interactions between these species and use theoretical models to examine the influence cell-cell interactions on spatially non-uniform microbial populations. Our results demonstrate that although interactions between species are key regulators of system behavior, only considering interactions between pairs of species is sufficient to predict ecosystem activity. Simulations demonstrate that activity at both the single-cell and population level would be strongly influenced by how microbes are distributed in space. These findings improve our understanding of how best to examine groups of microbes that coexist in environments such as soil, water, and the human body.

## Introduction

We are surrounded by complex communities of microbes, many that play a fundamental role in our everyday lives. Microbial ecosystems in nature are typically composed of hundreds or thousands of microbial species, heterogeneously distributed in space and time. Working together, these networks of microorganisms are critical in environmental remediation, food production, wastewater treatment, and human health and disease and there is great interest designing synthetic microbial ecosystems for new biotechnologies. Given the diversity of microbial ecosystems [1], there are many potential types of interactions between species, including all combination of positive, negative, and neutral interactions [2]. Understanding the properties of the community interaction network will help identify strategies to study and manipulate microbial networks. Developing a quantitative understanding of how ecosystems of microbes interact will be essential to predicting how microbial networks respond to environmental or biological changes and aid in designing synthetic communities with tailored functionality [3].

To quantify how interactions between species impact overall community function, previous experimental work has systematically measured pairwise interactions between species, such as crossfeeding between *E*. *coli* auxotrophs [4, 5] or natural isolates [6, 7]. Other work has inferred interactions between species from measurements of the population dynamics within a complex community [8–11]. These previous studies focused on pairwise interactions and the ability of pairwise interaction models to predict network function [2, 12]. Given the increase in data for biological interactions, there is great emphasis on the development of predictive models of biological networks, including constraint based, Boolean, and directed network models [13–18].

It is currently unclear how to accurately account for cell-cell interactions within models of microbial ecosystems and whether pairwise interactions are generally sufficient. Foster and Bell measured total productivity of subsets of microbial microcosms with up to 72 species, and no evidence for positive high-order interactions was observed [19]. In other biological contexts, measurements of interactions in neural or cytokine networks revealed pairwise interactions were dominant, although a few high-order combinations significantly altered patterns of cytokine activity [20, 21]. The impact of high-order interactions, between 3 or more components, have not been quantified in microbial ecosystems.

Here we examine the interaction network of a four-species microbial community through the systematic experimental measurement of multispecies interactions and a theoretical model. Using a fluorogenic indicator, we quantify the metabolic rate of different subsets of this community at a variety of species ratios. From these measurement, interactions coefficients are inferred that account for two to four-species interactions using a modified general Lotka-Volterra model [8]. We found that interactions between species do influence overall community activity. We examine the contribution of pairwise and higher interactions within the model community, and predict the impact of such interactions on community-level activity using a theoretical model. Although in the model community, overall activity is well described by pairwise model, theoretical results highlight the importance of the interaction network and specifically high-order interactions in spatially heterogeneous populations of cells.

## Results and Discussion

### Interactions between species set the overall metabolic rate of a four species microbial community

**Fig 1:**
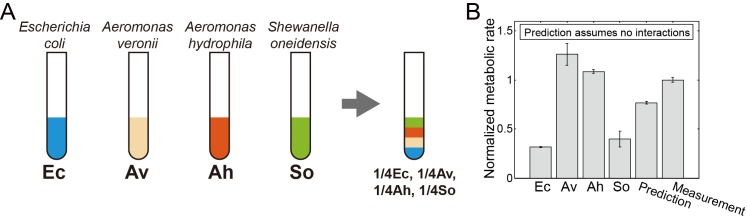
Metabolic activity of a 4-species community. **(A) The community contains four freshwater isolates, *Escherichia coli* (*Ec*), *Aeromonas veronii* (*Av*), *Aeromonas hydrophila* (*Ah*), and *Shewanella oneidensis* (*So*).** Species were grown separately and combined to measure the impact of multispecies interactions on the metabolic rate. (B) The overall metabolic rate of all four strains together was greater than the prediction made from measurements of the metabolic rate of individual strains, demonstrating that multispecies interactions contributed to the overall metabolic rate. Error bars show standard error. Metabolic rate has been normalized such that the 4-strain measurement has a value of 1.

The four strains that comprise the community were all isolated from freshwater environments, including three isolates from the Los Angeles area and the previously isolated *Shewanella oneidensis* MR-1 [22]. Strains were collected near the water surface and isolated on low strength LB plates. 16S rRNA sequencing has identified the strains as being closely related to *Escherichia coli* K-12, *Aeromonas veronii*, and *Aeromonas hydrophila*, see S8 Text for details. Throughout the paper these strains will be referred as *So*, *Ec*, *Av*, and *Ah* respectively, as shown in Fig 1A. We aim to elucidate the general properties of the interaction network within a microbial community by exhaustively measuring the output of all subsets of the community under a specific set of conditions. These four species were chosen based on viability under the same culturing conditions, no particular metabolic capabilities or potential for interactions were assumed.

To quantify the contribution of interactions between the species on metabolic rate, we first measured the metabolic rate of the four strains in isolation. Strains were grown in 5 mL scale cultures of low strength LB. After growth to an OD_600_ around 0.2, strains were transferred to a 96 wellplate. The metabolic rate was quantified using a fluorogenic assay for the presence of metabolic intermediates, the AlamarBlue assay containing the redox activity indicator compound resazurin [23–25]. Resazurin based assays have been used to quantify metabolism in a variety of bacterial and eukaryotic cells [26–32], making it a useful, universal indicator for metabolism in multispecies bacterial communities. Resazurin-based metabolic assays are based on the reduction of non-fluorescent resazurin to fluorescent resofurin by redox active compounds inside the cell. Although components of respiratory chain are known to reduce resazurin [33], multiple redox active compounds likely contribute to the fluorescent signal. Resofurin can also undergo a second, reversible redox reaction, forming a non-fluorescent compound [28], so care must be taken to ensure that the fluorescent signal is proportional to cell number and activity, as shown in S2 Text. Resazurin-based assays are amenable to high throughput measurements [29, 34], an advantage we leverage here for a comprehensive characterization of the four-species metabolic network. In initial measurements, the relative metabolic rates of the four species were determined, as shown in Fig 1B.

To determine the influence of multispecies interactions on ecosystem outputs, we compared the overall metabolic rate of a 4-species microbial community to the metabolic rate of each strain in isolation. If the species did not interact, the overall metabolic rate of 4 strains together would simply be the average metabolic rate. However when mixing the 4 species together the overall metabolic rate was 31% larger than the prediction made assuming no interactions, as shown in Fig 1B. This demonstrates that interactions between species significantly modulate the metabolic rate of one or more strains within the community. To further dissect the distribution of interactions within our community, we measured interactions between all combinations of species.

### Measuring the interaction between two species

**Fig 2:**
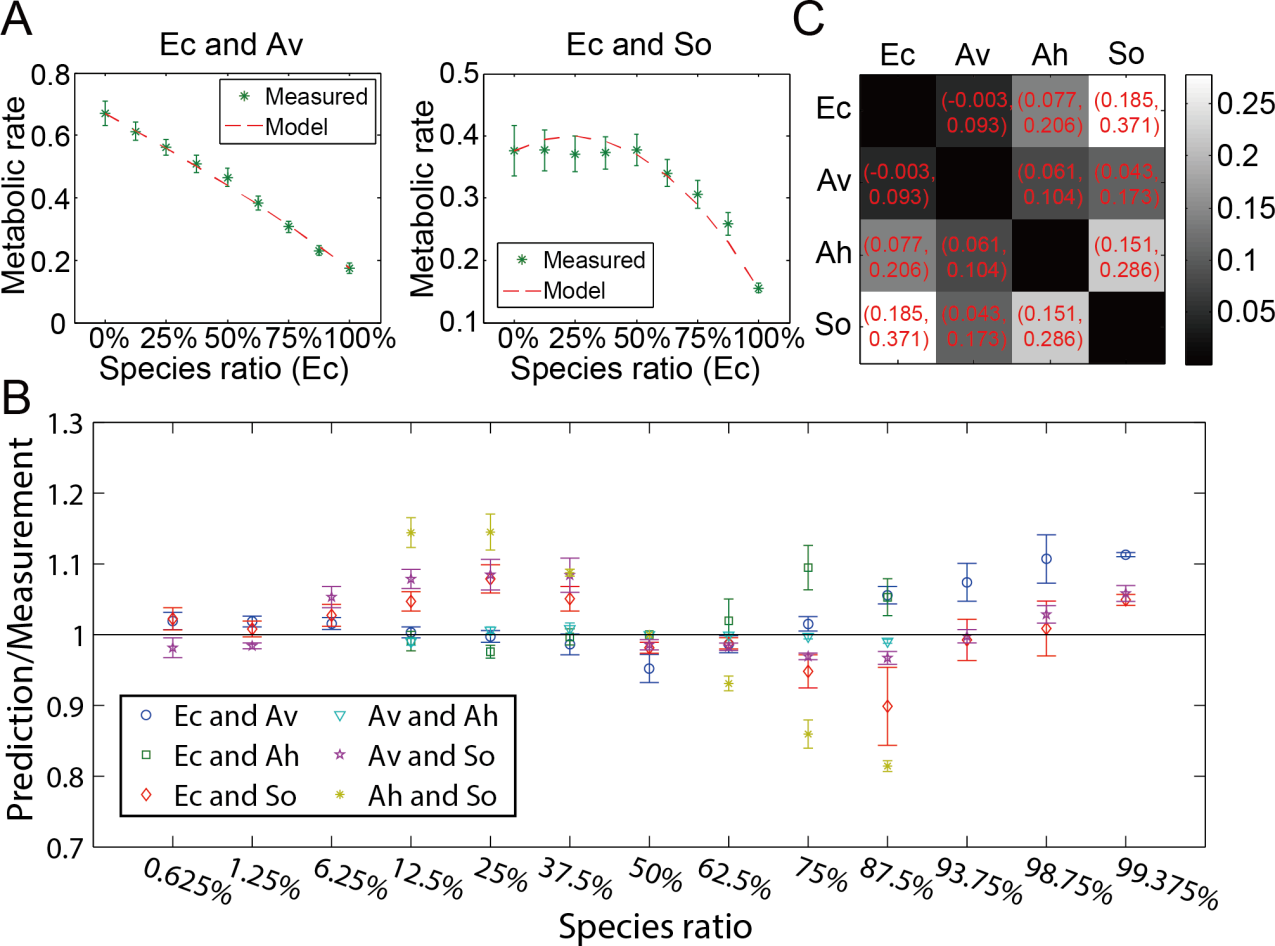
Pairwise interactions within the microbial community. (A) We measured metabolic rates at seven different ratios from 1/8 to 7/8 for the pair *Ec* and *Av* and the pair *Ec* and *So*. Interaction coefficients were extracted from the data and used to plot the best-fit model. (B) All pairwise combinations of the four strains were assayed. Predictions using solved interaction coefficients were compared to experimental measurements. The normalized strengths of interaction for all 6 pairwise combinations are shown in (C) for species ratio 1:1. Red numbers show confidence intervals at confidence level 95%. Error bars show standard errors of at least three measurements.

Pairwise interactions models are common to predict the activity of microbial networks. We measured the metabolic activity of two-species mixed cultures to determine if pairwise models could account for interactions within our microbial community (Fig 2). The strains were grown separately, mixed together for 30 minutes, and then metabolic activity was measured using the fluorogenic indicator. The ratio of the two species was varied between 1:7 and 7:1 to quantify how interactions between species depended upon the ratio of species. The metabolic rate was found to be linearly proportional to the number of cells measured, as shown in S2 Text. Metabolic activity of each species alone was measured to determine the baseline metabolic rate. The six sets of measurements for each pair of species shown in Fig 2A were taken on six different days, demonstrating the reproducibility of interactions.

To analyze how interactions between species determined the overall metabolic rate, we implemented a model in which the overall metabolic rate is modulated by an interaction parameter, as shown in Eq (1),

$$R\left(X,Y\right)=R\left(X\right)\cdot N_{x}/N_{total}+R\left(Y\right)\cdot N_{y}/N_{total}+i_{xy}\cdot p\left(N_{x},\;N_{y},\;N_{total}\right),$$ (1)

in which R(X), R(Y) and R(X,Y) are the average metabolic rates of species X, Y, and X and Y together respectively, i_xy_ is the pairwise interaction parameter that accounts for the increase or decrease of overall metabolic activity, N_x_, N_y_, and N_total_ are numbers of cells of species x, y, and the total number of cells in the single species control measurement, and p(N_x_, N_y,_) scales the interaction parameter based on the number of interacting cell. These General Lotka-Volterra models have been used previously [2]. The interaction coefficients can be positive or negative, such that the sum of the interactions did not result in non-physical negative metabolic rate. The metabolic rate (*R*) is proportional to the slope of fluorescence vs. time curve in experiments. We assume that p(N_x_, N_y,_) should be a function of N_x_, N_y_, and N_total_, as shown in Eq (2),

$$p\left(N_{x},\;N_{y},\;N_{total}\right)=(N_{x}/N_{total})\;\cdot \;(N_{y}/\left(N_{total}\right),$$ (2)

in which p is the product of ratios of species X and Y in the mixture. With the existence of p in the Eq (1), the interaction term is largest when equal numbers of species are present and will decay to zero as one of the populations dominates. Note that because we measure the overall metabolic output of combinations of species, the experiments cannot separate the individual impact of species X and Y and the impact of species Y on X. Our interaction term accounts for the overall change in activity due to species-species interactions.

The metabolic rate data was fit to determine the value of the interaction parameter, i_xy_. As shown in Fig 2A, the metabolic rate was measured in pairs of species over a range of ratios between the two species. As shown in Fig 2A, modeling pairwise interactions using Eq 1 agreed well with metabolic measurements of two strain mixtures over a range of species compositions.

The data in Fig 2A suggested that interactions for mixtures of species could be captured in a single interaction parameter. S3 Text shows the data for all species combinations, and most pairwise combinations are in close agreement with the prediction using a single interaction parameter. To determine whether the interaction strength was valid for all combination of the 4 species, in Fig 2B we plotted the ratio of prediction to measurement vs. species ratio for all 6 species combinations. We extended the species ratio to 0.625%, 1.25%, 6.25%, 93.75%, 98.75% and 99.375% for 3 combinations of species and found that the model fit data well even at these more extreme ratios of species.

We define the normalized interactions strength as the interaction term divided by the total metabolic rate when the species ratios are equal, which represents the maximum of the interaction term. In Fig 2C, we show the range of normalized interactions strengths in our system for all 6 pairwise combinations of *Ec*, *Av*, *Ah* and *So* at species ratio 1:1. The interaction coefficients were fit using all the data between species ratios of 0.625 to 99.375%. In our experiment, the first order normalized interaction strengths were all positive with values between 0.05 and 0.3.

### A predictive model incorporating higher-order multispecies interactions

**Fig 3:**
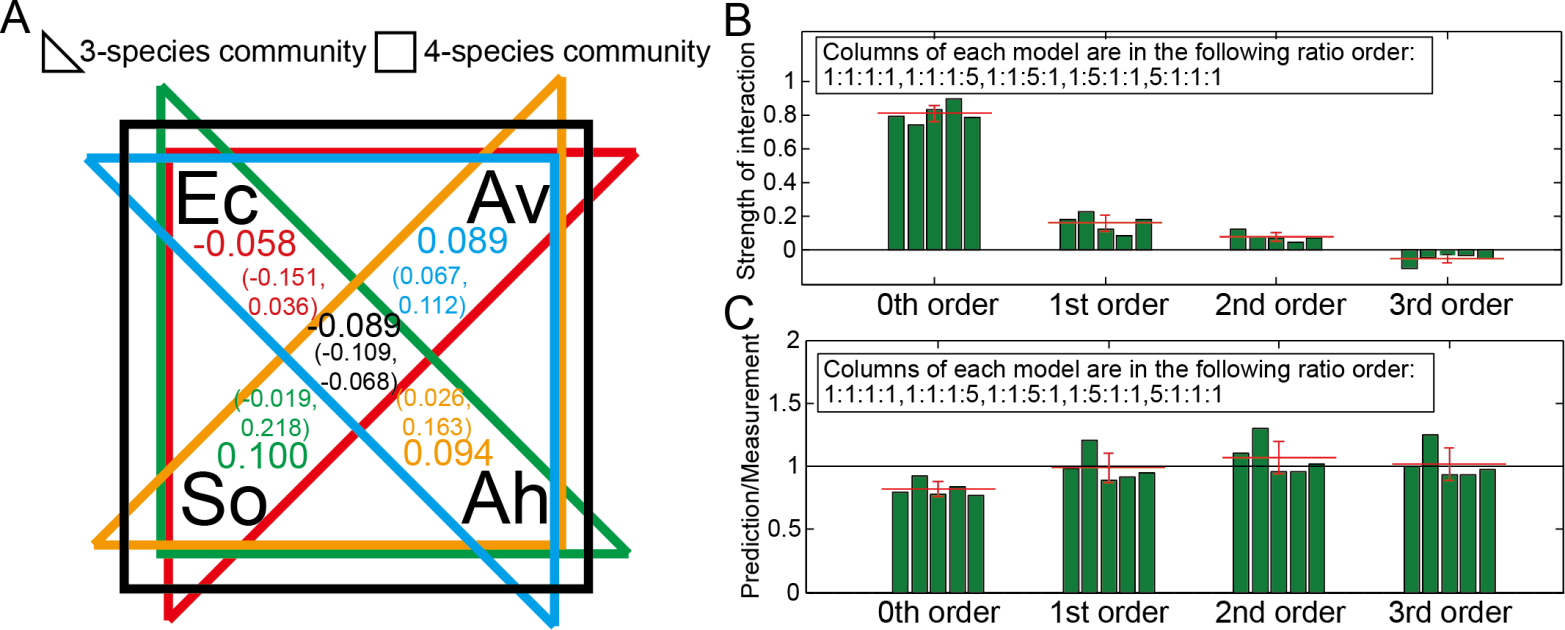
Accounting for higher-order interactions. (A) The proportions of 2^nd^ and 3^rd^ order interaction terms in 3 and 4 species microbial communities respectively. Interaction coefficients were calculated using Eqs 3 and 4 and experimental measurements of metabolic activity within subsets of the community. The 95% confidence interval is shown. (B) Proportions of different interaction terms in the 4-species community for different species ratios (Ec:Av:Ah:So). (C) Using all interaction coefficients derived from our data, we compared the experimental measurements of total activity of the 4-species community with even composition and with one member in excess. Predictions made assuming no interactions (0^th^ order model), 2-species interactions (1^st^ order model), and 2 and 3-species interactions (2^nd^ order model), and 2, 3, and 4-species interactions (3^rd^ order model). Error bars indicates standard deviations. Red lines indicate average values. The strength of interaction is normalized by dividing by the total metabolic rate.

Next the model was expanded to incorporate higher-order interactions between 3 or more species. The overall metabolic rate of the community is now,

$$R_{total}=\sum_{w=1}^{M}R_{x}p_{x}+\sum_{w=1}^{M}\sum_{x>w}^{M}i_{wx}p_{wx}+\sum_{w=1}^{M}\sum_{x>w}^{M}\sum_{y>x>w}^{M}i_{wxy}p_{wxy}+{i_{wxyz}p}_{wxyz},$$ (3)

in which M is the total number of species in the community, i_wx_ accounts for pairwise interaction between two species, i_wxy_ accounts for interactions between three species, and i_wxyz_ accounts for interactions between four species. This equation could be adapted to incorporate more higher-order terms. Building from the results of pairwise interactions, we approximate that higher-order interactions also dependent on the ratio of species. Similar to Eq 2, the scale factor p can be calculated from the following general form,

$$p=\prod_{w=1}^{M}\frac{N_{w}}{N_{total}},$$ (4)

where N_total_ is the total number of cells in the community. Analogous to finding the pairwise coefficients, measurements of the metabolic activity of three species combinations and Eqs 3 and 4 together with the pairwise interaction coefficients already measured were used to calculate the 4 second order coefficients. A single third order coefficient was calculated from experimental measurements of the 4 species community. The strengths of second and third order interaction terms in three- and four-species communities respectively are listed in Fig 3A.

Fig 3B shows in a four-species community, the proportions of all interaction terms in predicted overall metabolic rate for five different species ratios. The average contributions of 0^th^, 1^st^, 2^nd^, and 3^rd^ order interaction terms, shown as red lines, sharply decrease. The contribution of each term is calculated as the strength of interaction, defined as the sum of all interactions of a specific order divided by the total metabolic rate. For example the strength of the 2^nd^ order interactions would be,

$$\mathrm{Strength of interaction}=1/R_{\mathrm{total}}*\sum_{w=1}^{M}\sum_{x>w}^{M}\sum_{y>x>w}^{M}i_{wxy}\frac{N_{w}N_{x}N_{y}}{N_{total}^{3}}.$$ (5)

After extracting all interaction coefficients within the 4 species community, we compared the predictive ability of models incorporating different levels of interactions. Fig 3C compares measurements of the 4-species at different ratios of cells to versions of the model incorporating subsequently higher-order interaction terms. The 0^th^ order model gives us a metabolic rate that is under predicted, and adding first order coefficients greatly improves the accuracy of the prediction. Incorporating 3- and 4-species coefficients gives an accurate prediction, but is not an improvement over the 1^st^-order model. In the S4 Text we compare measurements to theory for a wider range of community composition. On average the ratio of prediction to measurements is 0.82±0.06 for the 0^th^ order model, 0.98±0.11 for the 1^st^ order model, 1.07±0.13 for the 2^nd^ order model, and 1.02±0.13 for the full 3^rd^ order model. The interaction network was built from the bottom up, fitting for low order coefficients from measurements of the minimum number of combined strains. We also analyzed the data by fitting to only the four-species data, as shown in S5 Text, both using a pairwise only model and the model incorporating high-order interactions described in Eq 3. Fitting only the four-species data resulted in an interaction network that did not accurately describe the activity of pairwise combinations of species.

### Multispecies interactions in spatially fragmented microbial systems

**Fig 4:**
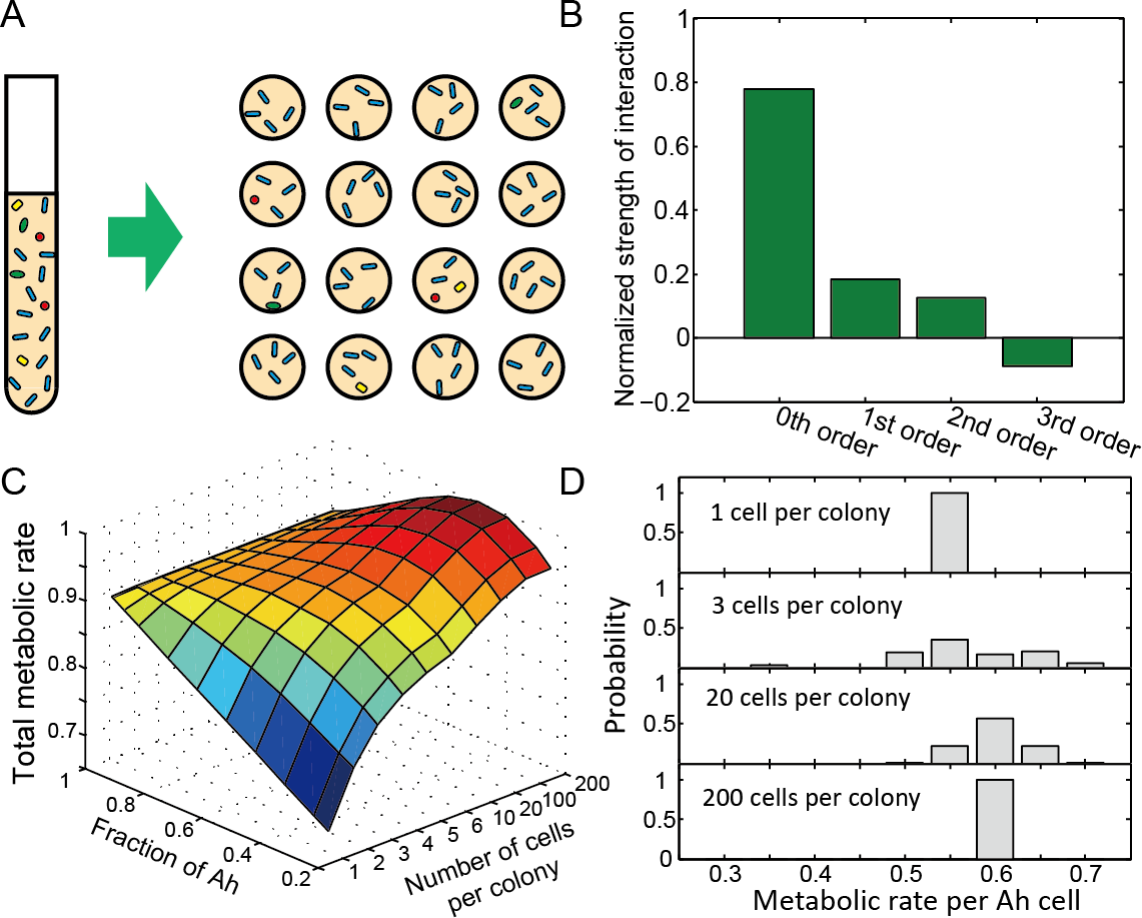
Simulations for a spatially fragmented multispecies microbial community. (A) The community is divided into microcolonies of variable size. (B) Relative contributions of different levels of interaction to the overall metabolic rate for the experimentally measured community. (C) Simulation results shown how the total metabolic activity of the system depends on the number of cells per microcolony and the community composition. The total metabolic rate has been normalized to 1. (D) Distributions of the metabolic rate of individual cells for microcolony sizes of 1, 3, 20 and 200. Metabolic activity was calculated using Eq 3, with basal metabolic rates of 0.16, 0.66, 0.57, 0.34 for Ec, Av, Ah, and So respectively. The fraction of Ah is 0.5 in (D).

Given the importance of interactions in setting overall activity levels of multispecies microbial communities, simulations were used to explore the experimentally measured interaction network in the context of a spatially structured population, as depicted in Fig 4A. Some natural microbial ecosystems have disperse, patchy distribution of cells [35, 36], potentially giving rise to many local neighborhoods of cells with a range of activity levels. If specific combinations are significant contributors to the overall activity level of the community, the size of these microcolonies and the evenness of the population could have significant consequences on the overall community activity. Agent-based models using Eqs 3 and 4 were used to explore the consequence of multispecies interactions in the limit of spatially isolated or non-interacting microcolonies.

A community incorporating 2-species, 3-species and 4-species interactions was simulated that contained the experimentally determined interactions parameters from Figs 2 and 3. Fig 4B shows the relative contribution of pairwise and higher-order interactions to the overall metabolic rate. The system contains 8,400 cells, unevenly distributed with a variable number of Ah cells and equal numbers of cell Av, So, and Ec making up the remainder of the population. The cells are randomly distributed into microcolonies such that each one has an equal number of cells. After distributing the cells, the activity of each group of cells is calculated using Eq 3 and assuming that interactions are local, i.e. restricted to neighbors within the same colony. The total activity of the community is the sum of the activity of all the cells in each micro-colony.

As shown in Fig 4C, the overall metabolic rate of the community is sensitive to how the species are distributed in space. Community activity increases with colony size as larger colonies allow the positive pairwise and three-species interactions. An even population distribution, not dominated by Ah, also leads to an increased metabolic rate as the positive interactions between the four strains are more likely to be sampled in each microcolony.

Group size also impacts the distribution of local activity levels. Fig 4D shows the activity of each microcolony in populations containing 50% of species Ah for microcolony sizes of 1, 3, 20, and 200 cells. We observe that for 1-cell microcolonies activity is low, as groups that are too small omit even 1^st^-order interactions. For small groups containing multiple cell types, the activity of individual cells broadens as a result of the variability of the composition of each microcolony. As microcolony size continues to expand, the average composition becomes more predictable, sampling all possible interactions, and single-cell activity levels are uniform. The distribution of activity levels in even this simple interaction network demonstrates how both the global species composition and the microscale distribution of cells can strongly influence local activity profiles. Local “hotspots” may be present in such populations, but only when populations are fragmented into small groups.

## Discussion

Here we measured the metabolic interactions within a four species community of microbes, to quantify the influence of pairwise and higher-order interactions on the overall metabolic rate of the community. In previous studies, pairwise interactions have been measured, including a large screen of *Streptomyces* species [7]. Similar to these previous studies, we found a range of interactions between the species, ranging from no interaction to strongly positive. Interestingly, no negative pairwise interactions were found in within the small set of species tried here, despite previous work finding that many pairwise interactions were negative [19]. One possible explanation is the use of dilute LB media, a complex media that may contain compounds that negatively impact the growth of some strains. Interactions with So were performed at 37°C, which is higher than its optimal growth temperature of 30°C [37]. The temperature stress on So may be related to the positive pairwise interactions with the other community members. Interactions between some species likely depend on cellular state and growth phase, and here we grew each species to exponential phase. We also examined metabolic rates after only 30 minutes of coculture, a time scale over which changes in species ratios are small, see S1 Text for growth rates. Our measurements capture changes in metabolic rate that occur on short timescales, and may not indicate the long term behavior of such systems, such as alteration of the growth environment by different types of cells. In multispecies communities long-term adaption can also change interaction networks, leading to unexpected and sometimes uncertain outcomes [38–40].

Quantitative measurements of higher-order interactions between these four species revealed that pairwise interactions dominated and were sufficient to predict community overall activity. When not taking any interactions into account, the predictions of the overall metabolic rate were off by more than 18%. Fig 3C shows that a pairwise model predicted the overall metabolic rate of the four species community within 2%, whereas adding the 3-species and 4-species coefficients, within the error of the measurement, did not improve predictions. However, this by no means denies the possible influences of higher-order interactions in other communities. The distribution of the magnitude of these high-order interactions within other, more complex communities would be valuable in determining how sensitive a community would be to changes in the community diversity. It is possible that even rare combinations of species may have evolved to strongly interact in natural microbial ecosystems. For a community with 100 species, there are in total >160,000 3-species combinations. The difficulty in measuring these interactions increases as communities become more complex, and it is unclear if strong interactions would be seen in even higher-order combinations of species. More work is needed to explore how much is gained by quantifying high order interactions in more complex settings.

Extending these findings to predict the activity within more complex multispecies microbial ecosystems will require a combination of predictive theoretical models and perhaps new experimental tools to quantify interactions. With the rapid development of nanofabrication technology, microfluidic devices are widely used for single-cell analysis [41, 42], allowing us to study multispecies interactions at the microsystem level such as suggested in Fig 4 and elsewhere [43, 44]. We implemented a model in Fig 4 to explore how the species evenness and the interaction network set the overall ecosystem outputs. For systems in which species are fragmented into small microcolonies, high-order interactions and microcolony size could play a dominant role in setting ecosystem outputs, especially in networks with strong high-order interactions such as for a toy network shown in S7 Text. Small groups also displayed a broader range of local activity levels. Given that a patchy distribution of cells has been observed in many natural communities [45–49], such “hotspot” microcolonies may be important drivers of function of some real microbial communities containing high-order interactions between species. Our results point for a need of new experimental methods to identify such species combinations in real systems.

## Materials and Methods

In experiments, all strains were taken from frozen glycerol stocks and grown overnight in 10% LB media (BD). The next day, we pipetted 5 to 150 μL from the suspension cultures into 3mL 10% LB media and grew them again for 3 to 4 hours such that all cultures simultaneously grew to a final optical density at 600 nm near 0.2. *Ec*, *Av*, and *As* were grown at 37°C in 10% LB media, while *So* was grown at 30°C. Cells were cultured at 5 mL scale and shaken at 200 rpm.

For measurements of metabolic activity, 100 μL of 10% LB media and 80 μL culture were pipetted into the wells of a 96-well microplate. The 80 μL suspension cultures could be single-species or multiple-species mixed to different ratios. The microplate was incubated at 37°C for 30 minutes to allow microbial communities to interact. Finally, we pipetted 20 μL of the metabolic indicator AlamarBlue (Thermo Scientific) into each well and measured the fluorescence change using a well-plate reader. Fluorescence was measured at excitation and emission wavelengths of 560 to 590 nm, and a media-only control was used to account for background fluorescence. S6 Text shows that during the measurement, the OD_600_ of cultures increased, as expected given the doubling times reported in S1 Text.

For two-species combinations, we measured different ratios from 1:7 to 7:1. For three-species combinations, we measured 6:1:1, 1:6:1, 1:1:6, 4:2:2, 2:4:2, 2:2:4, 2:3:3, 3:2:3, 3:3:2. For four-species combinations, we measured 1:2:2:3, 1:2:3:2, 1:3:2:2, 2:1:2:3, 2:1:3:2, 3:1:2:2, 2:2:1:3, 2:3:1:2, 3:2:1:2, 2:2:3:1, 2:3:2:1, 3:2:2:1, 2:2:2:2, 1:1:1:5, 1:1:5:1, 1:5:1:1, 5:1:1:1. All combinations were repeated at least three times on different days. The interaction coefficient from each day was solved separately and interaction coefficients from different days were used to calculate the mean, standard error, and confidence interval of each interaction parameter. Interactions coefficients in Eq 3 were solved using Solver in excel (GRG Nonlinear solving method) to minimize the sum of differences between predictions and measurements of all ratios. In higher-order models, we applied the same method to solve for higher-order interaction coefficients, using lower-order interaction coefficients solved previously in experiments involving fewer species.

## Supporting Information

S1 Text Optical densities (OD 600) of cell cultures growing in 10% LB. (DOCX)

S2 Text Metabolic rate is linearly proportional to the cell density. (DOCX)

S3 Text Predicted and measured metabolic rates for species ratios from 1:7 to 7:1 for all 6 2-species combinations. (DOCX)

S4 Text Comparison of predictions to measurements and contribution of each interaction term for a wide rage of species ratios. (DOCX)

S5 Text Interaction parameters fit using only the data from 4-species experiments. (DOCX)

S6 Text Measurements of absorbance at 600 nm during the AlamarBlue assay indicate cell growth. (DOCX)

S7 Text Simulations for spatially fragmented multispecies microbial community. (DOCX)

S8 Text 16s rRNA Sequences. (DOCX)

S9 Text Calculation of the confidence intervals for the interaction parameters. (DOCX)
