# Supplementary material for: The Contribution of High-Order Metabolic Interactions to the Global Activity of a Four-Species Microbial Community
Source: PLoS Comput Biol. 2016 Sep 13;12(9):e1005079. doi: 10.1371/journal.pcbi.1005079 (PMC5021341; doi:10.1371/journal.pcbi.1005079)
Supplement: S1 Text — (DOCX) [file pcbi.1005079.s001.docx]

The four strains we used in our experiment were from isolated from freshwater environments. Strains *Av* and *Ah* were isolated from the top 5 cm of the lake in MacArthur Park in Los Angeles, CA (34.059°N, 118.278°W). *Ec* was isolated from a freshwater pond on the campus of the California Institute of Technology in Pasadena, CA (34.138° N 118.125° W), and was indistinguishable from the common lab strain *Escherichia coli* K-12 by 16S rRNA sequencing. Strains were preserved in 50% glycerol stock solutions at -80 ^o^C.


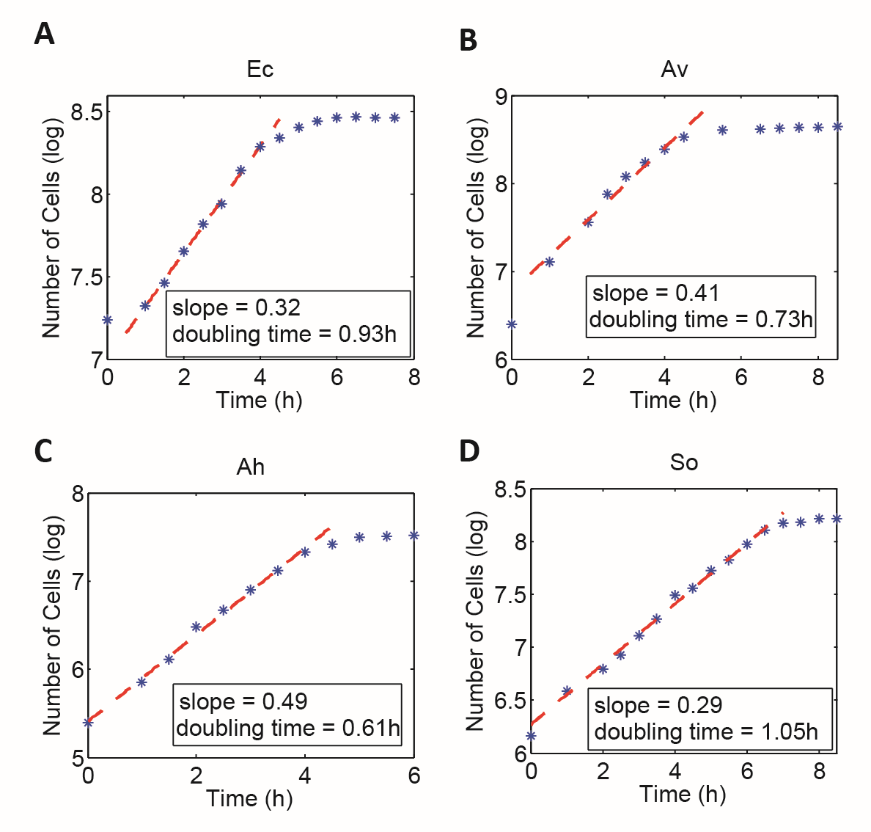


Figure S1: **Optical densities (OD 600) of cell cultures growing in 10% LB.** Dashed line shows linear fit to growth curve during exponential growth. *Ec*, *Av*, and *Ah* were grown at 37 °C in 10% LB media. *So* was grown at 30 °C in 10% LB media.
