# Supplementary material for: The Contribution of High-Order Metabolic Interactions to the Global Activity of a Four-Species Microbial Community
Source: PLoS Comput Biol. 2016 Sep 13;12(9):e1005079. doi: 10.1371/journal.pcbi.1005079 (PMC5021341; doi:10.1371/journal.pcbi.1005079)
Supplement: S2 Text — (DOCX) [file pcbi.1005079.s002.docx]

**Measuring Interactions**

For two-species combinations, we measured different ratios from 1:7 to 7:1. For three-species combinations, we measured 6:1:1, 1:6:1, 1:1:6, 4:2:2, 2:4:2, 2:2:4, 2:3:3, 3:2:3, 3:3:2. For four-species combinations, we measured 1:2:2:3, 1:2:3:2, 1:3:2:2, 2:1:2:3, 2:1:3:2, 3:1:2:2, 2:2:1:3, 2:3:1:2, 3:2:1:2, 2:2:3:1, 2:3:2:1, 3:2:2:1, 2:2:2:2, 1:1:1:5, 1:1:5:1, 1:5:1:1, 5:1:1:1. Species by themselves have also been measured to obtain the original metabolic rates. Every combination had been repeated at least three times.

The absorbance and fluorescence were tracked for one hour in the plate reader. The slope of the first 10 minutes of fluorescence versus time is used to calculate the metabolic rate at t=0. The measurements were adjusted to the single-species control, thus taking into account the change in cell number relative to the single-species measurement. Figure A shows that for the range of cell densities used in the experiments, the metabolic rate, as measured using the AlamarBlue assay, is linearly proportional to the number of cells for each of the four species in the community. All cultures were grown to an optical density at 600 nm of approximately 0.2 for all experiments.


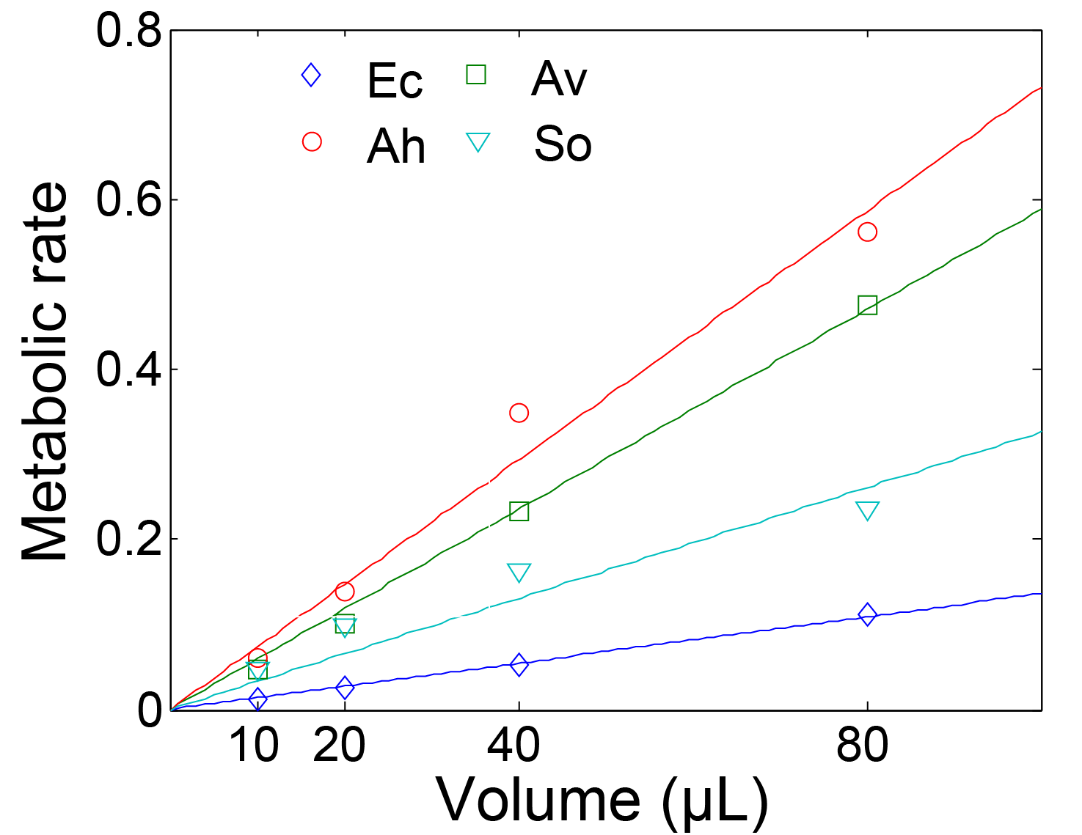


Figure S2: **Metabolic rate is linearly proportional to the cell density.** Different volumes of cell cultures from 10 to 80 μL were diluted to 180 μL with 10% LB media. After incubating at 37°C for 30 minutes, 20 μL of AlamarBlue was added and the metabolic rates were measured by using a well-plate reader. This graph shows the metabolic rate is proportional to the cell density. Best-fit lines are constrained to pass through the origin.
