# Supplementary material for: The Contribution of High-Order Metabolic Interactions to the Global Activity of a Four-Species Microbial Community
Source: PLoS Comput Biol. 2016 Sep 13;12(9):e1005079. doi: 10.1371/journal.pcbi.1005079 (PMC5021341; doi:10.1371/journal.pcbi.1005079)
Supplement: S3 Text — (DOCX) [file pcbi.1005079.s003.docx]

Equations 1 and 3 from the main text were used to solve for interaction coefficients. Fig. A shows that for most pairwise combinations, our model is in good agreement with experimental measurements.


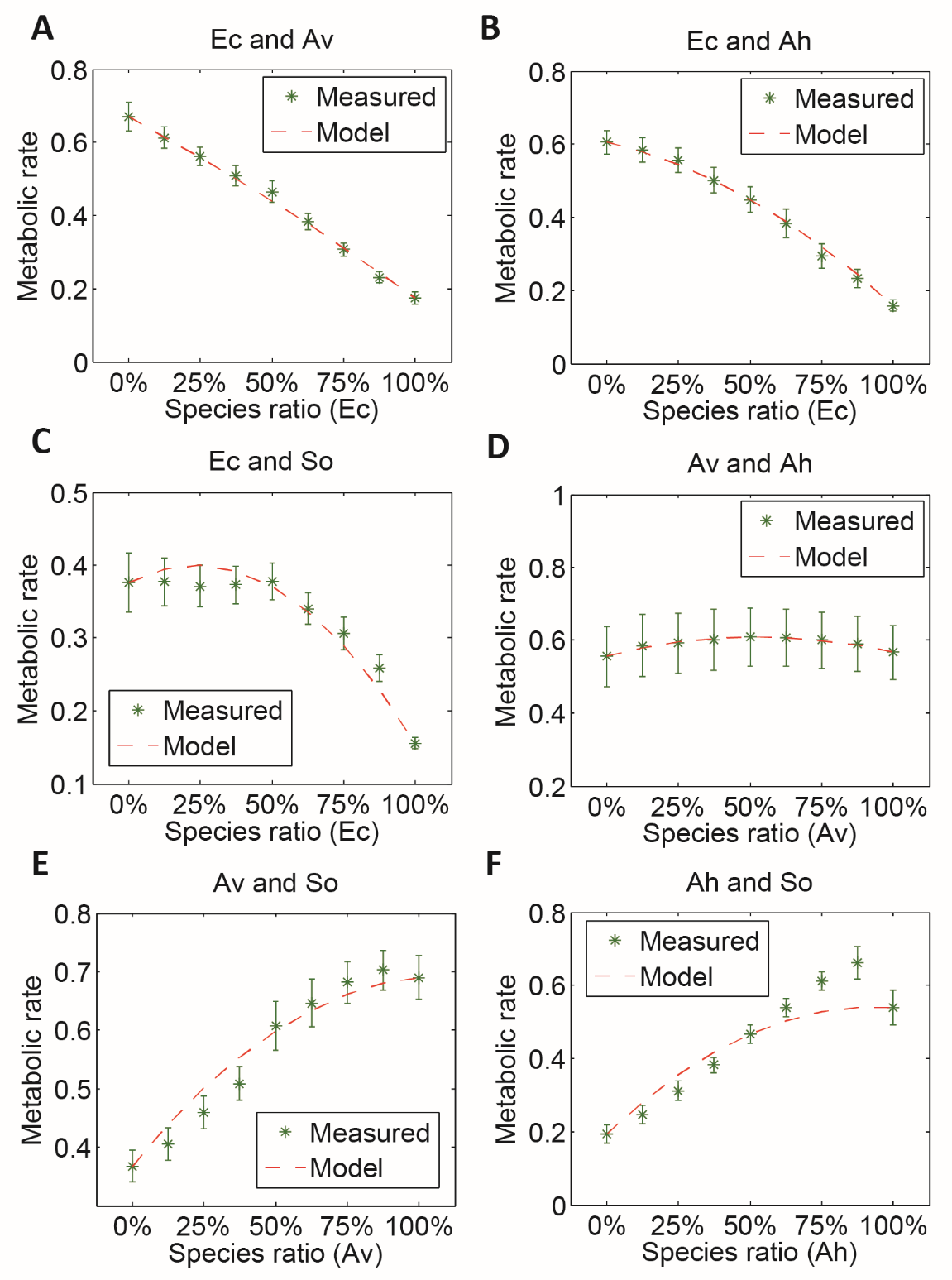


Figure S3: **Predicted and measured metabolic rates for species ratios from 1:7 to 7:1 for all 6 2-species combinations.** Interaction coefficients were extracted from the data and used to plot the predictions lines, which are red. Data in A is also shown in Figure 2 of the main text. Each data point was measured on at least three different days and error bars show standard errors.
