# Supplementary material for: The Contribution of High-Order Metabolic Interactions to the Global Activity of a Four-Species Microbial Community
Source: PLoS Comput Biol. 2016 Sep 13;12(9):e1005079. doi: 10.1371/journal.pcbi.1005079 (PMC5021341; doi:10.1371/journal.pcbi.1005079)
Supplement: S4 Text — (DOCX) [file pcbi.1005079.s004.docx]

Fig. A compares predictions of the 4-species community with 0^th^ order (assuming no interactions among species), 1^st^ order, 2^nd^ order, and 3^rd^ order models for 17 different ratios. The 0^th^ order model gives us a metabolic rate that is under predicted, and adding second order coefficients greatly improves the predictions. Fig. B shows in the four-species community, the proportions of all interaction terms in predicted overall metabolic rate for 17 different ratios. The average contributions of 0^th^, 1^st^, 2^nd^, and 3^rd^ order interaction terms, shown as red lines, sharply decrease.


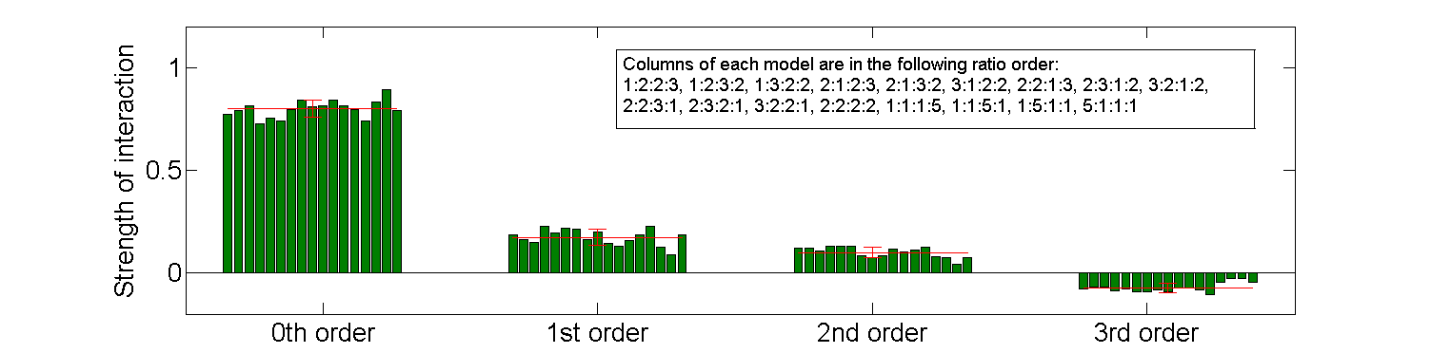


Figure S4.1: **Comparison of predictions to experimental measurements of total activity of the 4-species community over a wide range of species ratios.** Predictions made assuming no interactions (0^th^ order model), 2-species interactions (1^st^ order model), and 2 and 3-species interactions (2^nd^ order model), and 2, 3, and 4-species interactions (3^rd^ order model). Error bars indicates standard deviations. Red lines indicate average values.


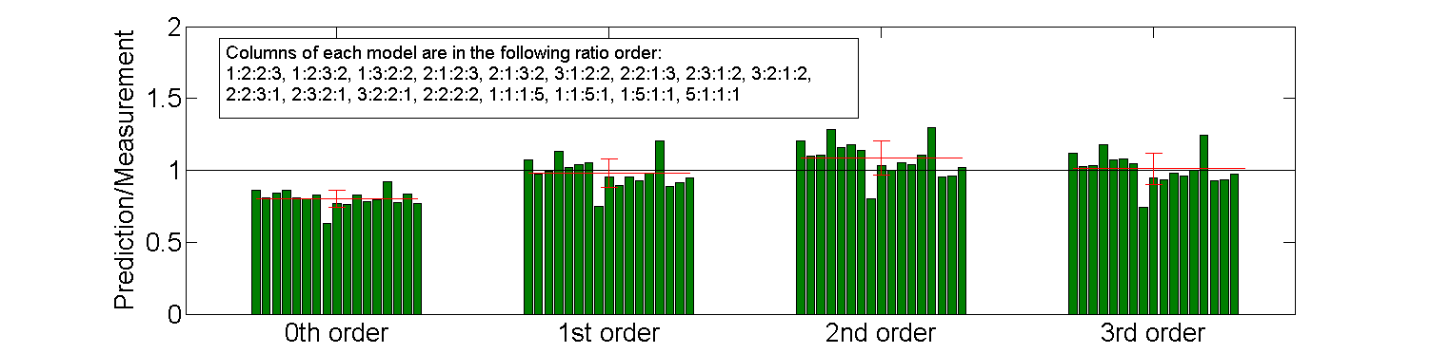


Figure S4.2: **The contribution of each interaction term in Equation in the 4-species community for different species ratios.** Error bars indicates standard deviations. Red lines indicate average values. Strengths of interaction were calculated as in Figure 3B.
