# Supplementary material for: The Contribution of High-Order Metabolic Interactions to the Global Activity of a Four-Species Microbial Community
Source: PLoS Comput Biol. 2016 Sep 13;12(9):e1005079. doi: 10.1371/journal.pcbi.1005079 (PMC5021341; doi:10.1371/journal.pcbi.1005079)
Supplement: S5 Text — (DOCX) [file pcbi.1005079.s005.docx]

Interaction parameters were also fit using only the data from 4-species experiments. The results of these fits are shown in Figures A and B. Although interaction parameters were found to be in agreement with the 4-species measurements, the pairwise interaction parameters were different than those calculated in the main text and were poorly fit to pairwise measurements of activity.


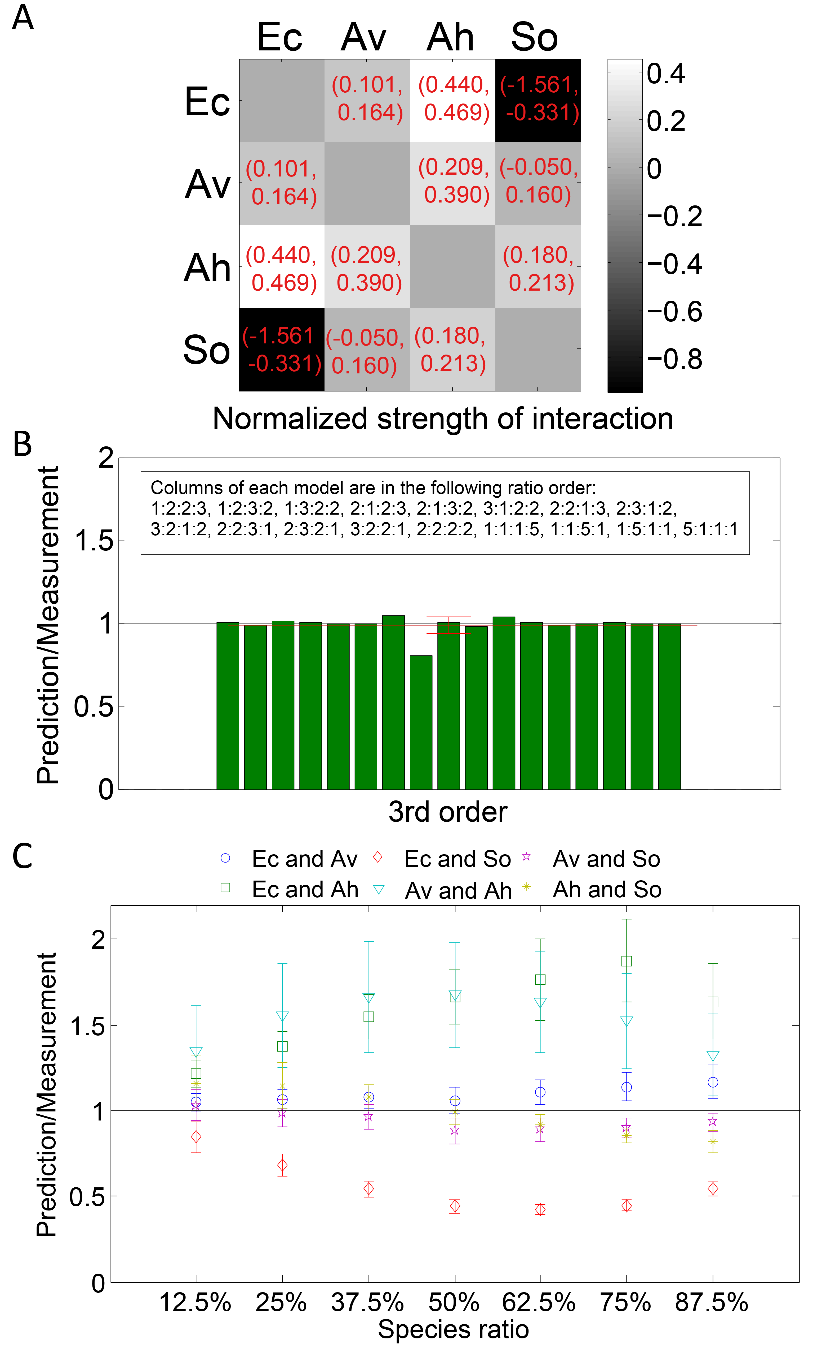


Figure S5.1: **All interaction parameters fit using only the data from 4-species experiments.** (A) Pairwise, 3-species, and 4-species interaction parameters were fit using only the data from 4-species experiments. The normalized strengths of pairwise interactions are shown for each pair of species. Red numbers show confidence intervals at confidence level 95%. (B) Using the full set of interaction parameters, predictions were in agreement with measurements of the metabolic activity of the 4-species community. (C) Predictions made using the pairwise interaction coefficients shown in A are not in agreement with 2-species measurements of activity.


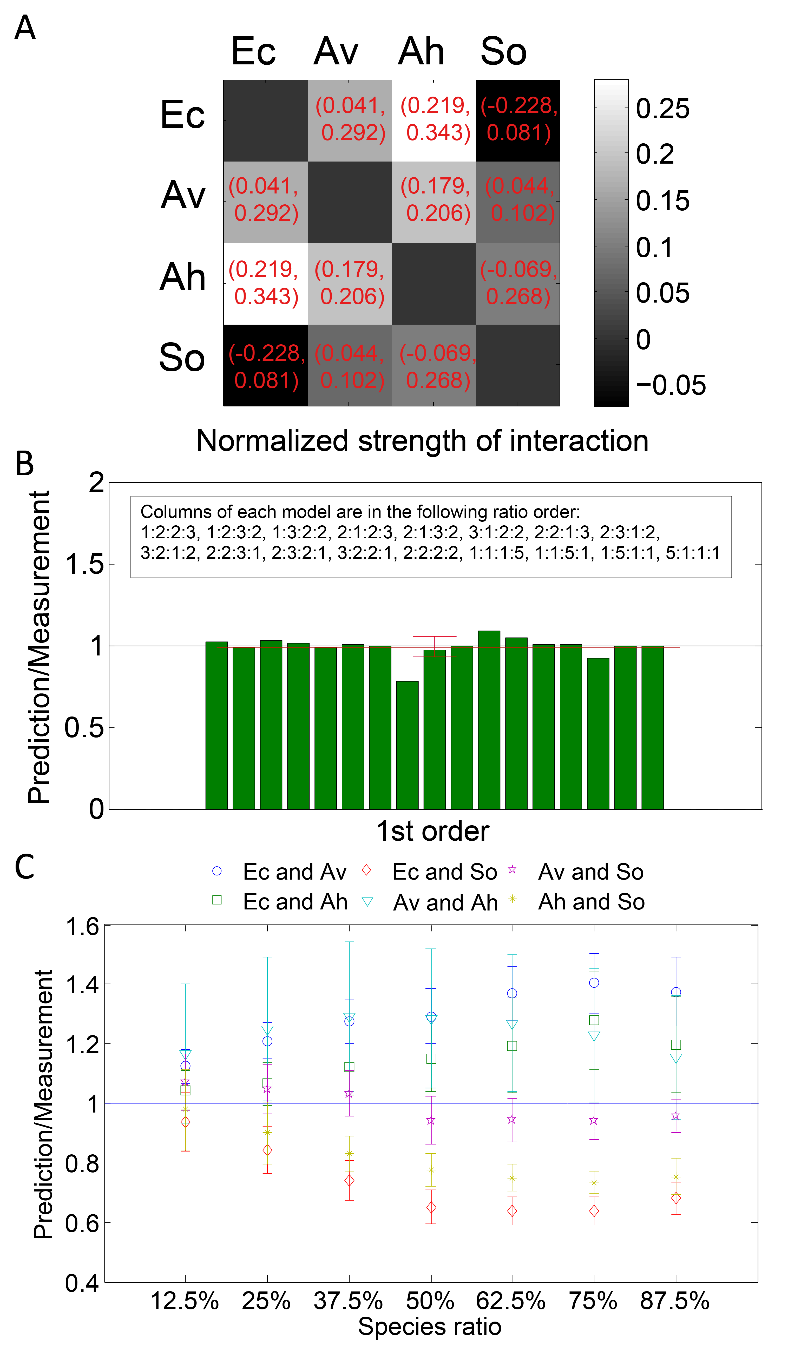


Figure S5.2: **Pairwise parameters fit using only the data from 4-species experiments.** (A) Pairwise parameters were fit using only the data from 4-species experiments, assuming no higher-order interactions. The normalized strengths of pairwise interactions are shown for each pair of species. Red numbers show confidence intervals at confidence level 95%. (B) Using the full set of interaction parameters, predictions were in agreement with measurements of the metabolic activity of the 4-species community. (C) Predictions made using the pairwise interaction coefficients shown in A are not in agreement with 2-species measurements of activity.
