# Supplementary material for: The Contribution of High-Order Metabolic Interactions to the Global Activity of a Four-Species Microbial Community
Source: PLoS Comput Biol. 2016 Sep 13;12(9):e1005079. doi: 10.1371/journal.pcbi.1005079 (PMC5021341; doi:10.1371/journal.pcbi.1005079)
Supplement: S6 Text — (DOCX) [file pcbi.1005079.s006.docx]

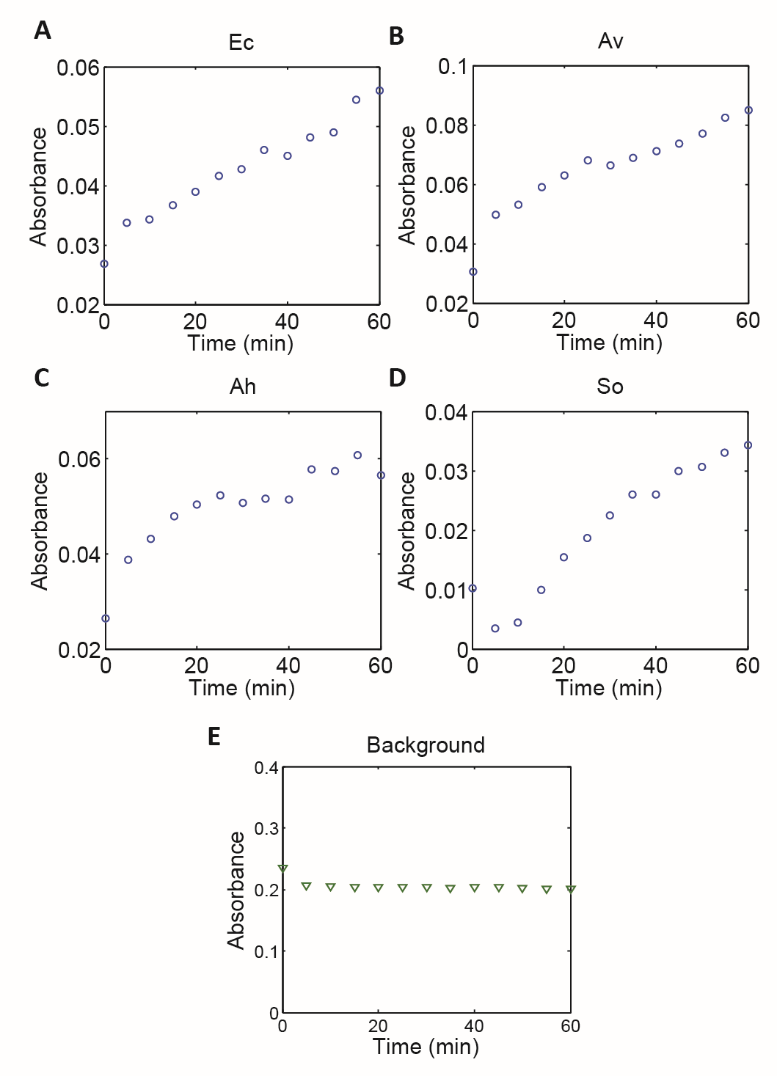


Figure S6: **Measurements of absorbance at 600 nm during the AlamarBlue assay indicate cell growth.** (E) During the assay the no cell negative control measurement remained constant.
