# Supplementary material for: The Contribution of High-Order Metabolic Interactions to the Global Activity of a Four-Species Microbial Community
Source: PLoS Comput Biol. 2016 Sep 13;12(9):e1005079. doi: 10.1371/journal.pcbi.1005079 (PMC5021341; doi:10.1371/journal.pcbi.1005079)
Supplement: S7 Text — (DOCX) [file pcbi.1005079.s007.docx]

**
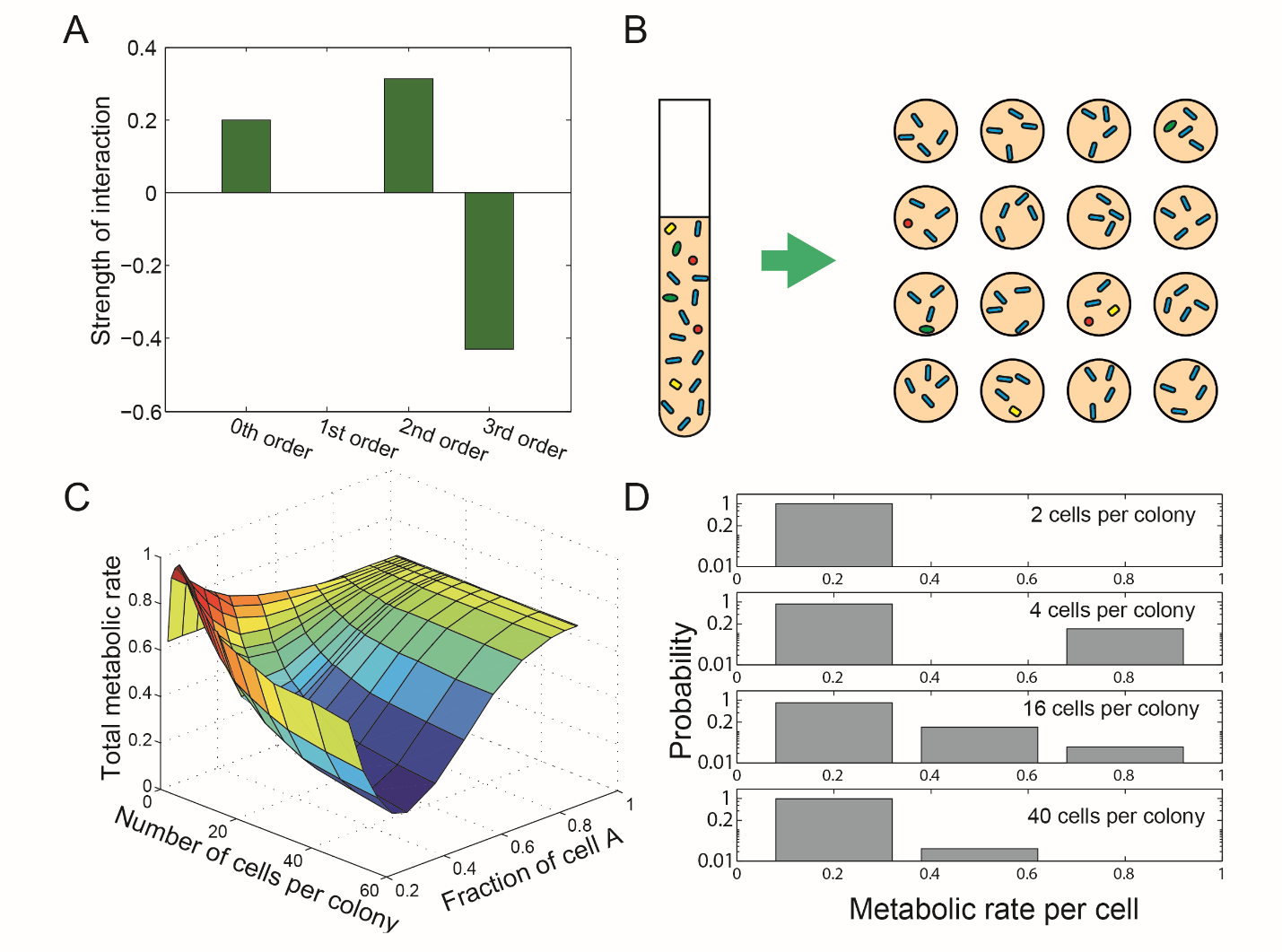
**

**Figure S7: Simulations for spatially fragmented multispecies microbial community.** (A) A theoretical 4-species microbial community with a positive 3-species interaction and a negative 4-species interaction. (B) The community is spatially fragmented into microcolonies of variable size. (C) Simulation result shown how the total metabolic activity of the system depends on the number of cells per microcolony and the community composition. The total metabolic rate has been normalized to 1. (D) Distributions of the metabolic rate of individual cells for microcolony sizes of 2, 4, 16, and 40. Metabolic activity was calculated using equation 3, with basal metabolic rates of 0.2 for all species, i_BCD_ = 20, and i_ABCD_ = -110. All other interaction terms were 0. The fraction of A is 0.5.
