# Supplementary material for: The Contribution of High-Order Metabolic Interactions to the Global Activity of a Four-Species Microbial Community
Source: PLoS Comput Biol. 2016 Sep 13;12(9):e1005079. doi: 10.1371/journal.pcbi.1005079 (PMC5021341; doi:10.1371/journal.pcbi.1005079)
Supplement: S9 Text — (DOCX) [file pcbi.1005079.s009.docx]

The 95% confidence intervals of interaction parameters were calculated as the following:

$(\bar{x}-1.96\cdot\frac{\sigma}{\sqrt{n}} , \bar{x}+1.96\cdot\frac{\sigma}{\sqrt{n}})$,

where $\bar{x}$ is the average interaction parameter from at least 3 measurements, $\sigma$ is the standard deviation of the sample, and *n* is the number of measurement.
